# Sacrococcygeal Chordoma: A Diagnostic Challenge

**DOI:** 10.7759/cureus.88799

**Published:** 2025-07-26

**Authors:** Maroua Slouma, Sameh Achoura, Samar Zarati, Hichem Ammar, Karima Tlili

**Affiliations:** 1 Pain Treatment Center, Rabta Hospital, Tunis, TUN; 2 Neurosurgery, Military Hospital of Instruction of Tunis, Tunis, TUN; 3 Family Medicine, Rabta Hospital, Tunis, TUN; 4 Pathology, Military Hospital of Instruction of Tunis, Tunis, TUN

**Keywords:** cauda equina syndrome (ces), chordoma, pancytokeratin, s100 protein, sacrococcygeal chordoma

## Abstract

Chordomas are rare, aggressive malignancies arising from notochordal remnants that mainly affect the sacrococcygeal region. Their insidious symptoms often delay the diagnosis. We reported a case of chordoma, emphasizing clinical, histological, and radiological features, and highlighting the diagnostic challenges.

We present the case of a 73-year-old woman with progressive low back pain, bilateral thigh radiation, and new-onset urinary and bowel dysfunction culminating in cauda equina syndrome. Neurologic assessment demonstrated preserved lower extremity motor strength (5/5) and symmetric reflexes but identified saddle hypoesthesia (S3-S5 dermatomes). Spine MRI revealed a large sacrococcygeal lesion with T2 hyperintensity, osteolytic destruction, compressing nerve roots, displacing the rectum, and infiltrating pelvic muscles. The patient underwent surgery with near-total resection (R1 margin due to sphincter preservation). Histopathology findings confirmed the diagnosis of chordoma by showing vacuolated physaliphorous cells in myxoid stroma with positivity of panCK and S100. Adjuvant proton therapy was recommended, but the patient declined. During the follow-up, she regained bowel function and pain relief.

In this case, sacrococcygeal chordoma was diagnosed at an advanced stage following the onset of neurological deficits, necessitating urgent surgical intervention. MRI was pivotal for both diagnosis and preoperative planning. Immunohistochemistry provided definitive pathological confirmation. Sphincter-preserving resection, in our patient, successfully maintained bowel function. Given our R1 resection margin, long-term surveillance remains crucial due to the high risk of local recurrence.

## Introduction

Chordoma is a rare malignant bone tumor that represents approximately 1-4% of all primary bone malignancies, with approximately 50% of cases localized to the sacrococcygeal region [1,2]. Chordoma usually presents as a slow-growing, expansile intraosseous tumor that progressively erodes the bony cortex and invades adjacent soft tissues [2]. Initially misclassified as cartilaginous tumors, chordomas were later identified as neoplasms of notochordal origin [1]. Recent research posits that they arise from persistent notochordal remnants. However, this theory remains debated, as soft tissue chordomas have been reported in locations devoid of known notochordal remnants, thereby challenging conventional etiological theories and raising the possibility of alternative origins and tumorigenic mechanisms [3]. Genetic alterations affecting the prognosis of chordoma have also been reported [4].

Patients with chordomas often have an indolent course with non-specific symptoms, leading to delayed diagnosis. Clinical manifestations are various and depend on the location of the chordoma. Patients with skull base chordomas often complain of headaches or cranial neuropathy, while those with spinal chordomas may present with axial pain, myelopathy, radiculopathy, or bowel/bladder dysfunction due to spinal cord or nerve compression.

Histologically, they are characterized by large vacuolated “physaliphorous” cells embedded within a myxoid stroma. Therapeutic management of chordoma is usually based on aggressive surgical resection, which can be associated with adjuvant radiation therapy and adjuvant chemotherapy in refractory cases.

We reported a case of chordoma, emphasizing clinical, histological, and radiological features, to underscore diagnostic challenges and management considerations.

## Case presentation

A 73-year-old woman presented with a six-month history of progressive, severe low back pain radiating bilaterally to the anteromedial thighs, characterized by sharp, burning pain exacerbated by standing or walking. Over the preceding month, she developed new-onset urinary incontinence and constipation, with no history of trauma, constitutional symptoms, or recent infections. Physical examination revealed severe functional impairment (inability to stand or walk), paravertebral muscle spasm, and focal lumbosacral tenderness with markedly restricted range of motion. Neurologic assessment demonstrated preserved lower extremity motor strength (5/5) and symmetric reflexes but identified saddle hypoesthesia (S3-S5 dermatomes). Pelvic examination showed no masses or tenderness, with normal passive hip mobility, and she remained afebrile. The constellation of saddle anesthesia, bowel and bladder dysfunction, and a presacral mass raises concern for cauda equina syndrome (CES) secondary to neoplastic or compressive pathology, necessitating urgent imaging.

**Figure 1:**
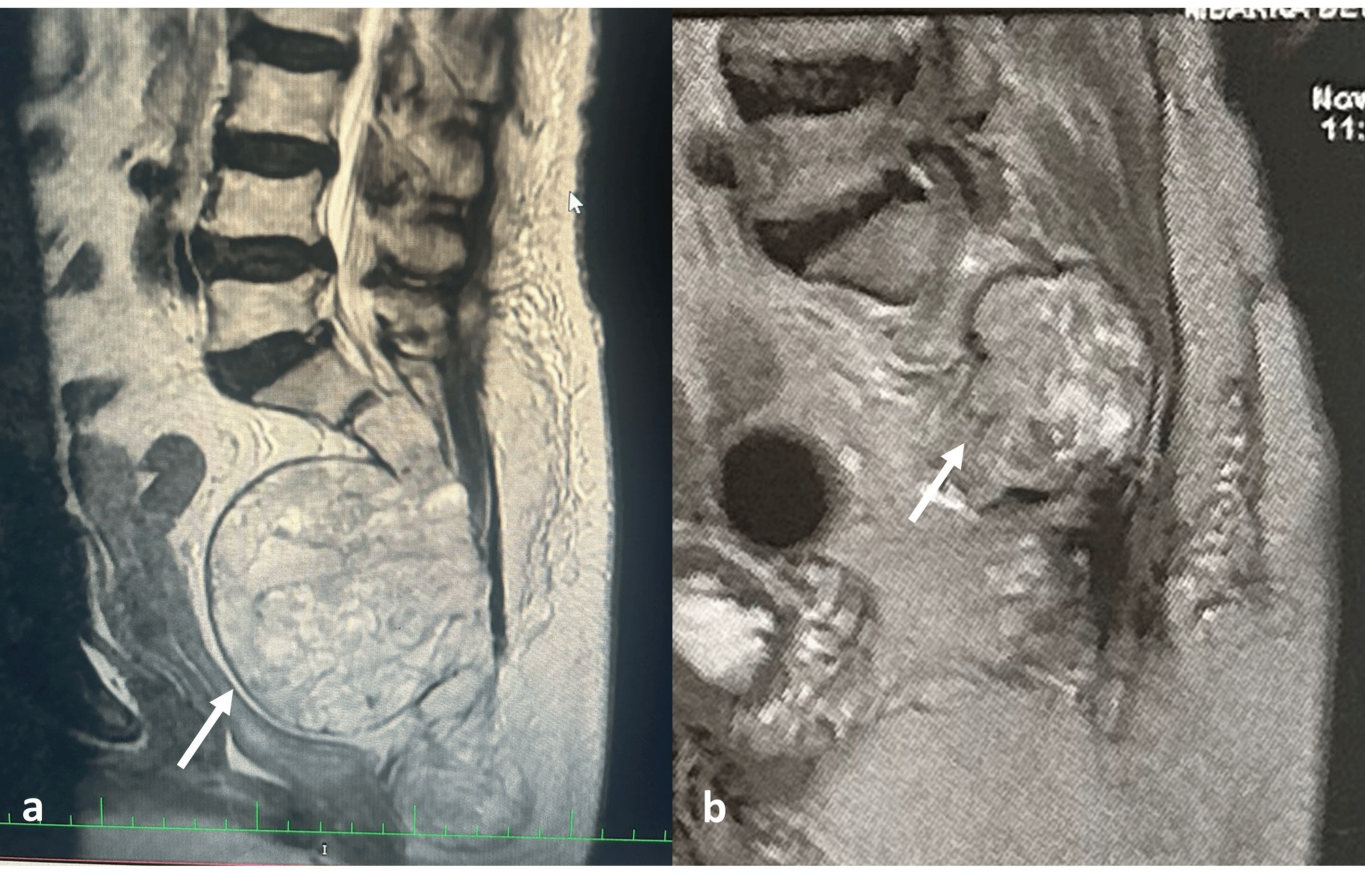
MRI findings (a) Preoperative sagittal T2-weighted MRI showing a large, well-circumscribed, hyperintense mass in the sacrococcygeal region, measuring 108 × 88 mm (arrow). The lesion causes extensive osteolytic destruction of the sacrum and coccyx, posterior compression of the cauda equina, anterior displacement of the rectum, and infiltration of adjacent soft tissues. (b) Postoperative sagittal T2-weighted MRI showing subtotal resection of the lesion (arrow) with decompression of the thecal sac and reduction of mass effect on adjacent structures.

Lumbosacral MRI identified a large, well-circumscribed sacrococcygeal mass (108 × 88 mm in axial dimensions). The lesion appeared hypointense on T1-weighted sequences and markedly hyperintense on T2-weighted and short tau inversion recovery (STIR) imaging, with heterogeneous post-gadolinium enhancement. Extensive osteolytic destruction of the sacrococcygeal bone was evident, accompanied by triplanar mass effect: posteriorly compressing and encasing the cauda equina nerve roots, anteriorly displacing the rectum, and laterally infiltrating the iliococcygeus and piriformis muscles (Figure 1a). The combination of midline sacrococcygeal localization, lytic bone destruction, T2 hyperintensity, and peripheral enhancement was highly suggestive of the diagnosis of sacrococcygeal chordoma.

The patient underwent urgent neurosurgical intervention. An inverted U-shaped cutaneous incision was centered over the sacral region, providing optimal exposure of the tumor. The tumor was carefully dissected, with its superior pole exposed at the level of the spinal canal. Intratumoral nerve roots were identified, showing clear infiltration by the tumor mass. The tumor margins were systematically dissected from paraspinal muscles, sacral ligamentous attachments, and the remaining inferior portion of the coccyx. Near-total tumor resection was achieved, including decompression of the intracanalicular component. A small portion of the tumor adherent to the anal sphincter complex was intentionally preserved to maintain continence. The surgical procedure lasted six hours.

**Figure 2:**
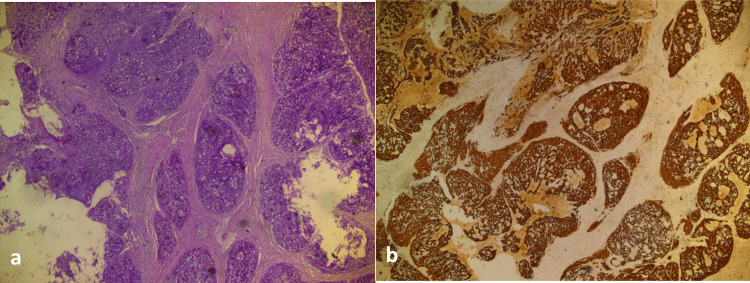
Histopathological examination (a) Histopathological examination showing a lobular growth pattern composed of trabeculae, cords, and solid nests of neoplastic cells with vacuolated cytoplasm and moderately atypical nuclei, embedded within an abundant myxoid stroma (H&E stain). (b) Immunohistochemical staining demonstrating expression of pan-cytokeratin (panCK) by the tumor cells.

Histopathological examination demonstrated a lobular growth pattern composed of trabeculae, cords, and solid nests of neoplastic cells featuring vacuolated cytoplasm and moderately atypical nuclei, embedded within an abundant myxoid stroma (Figure 2a). The tumor cells showed co-expression of pan-cytokeratin (pan-CK) (Figure 2b), supporting the diagnosis of chordoma.

Adjuvant proton beam therapy was recommended for the residual tumor. However, the patient declined this treatment option. During follow-up, the patient reported significant pain relief and restoration of baseline bowel function. At her 24-month follow-up, the patient reported chronic tenesmus, likely secondary to partial sacral nerve compromise during the sphincter-preserving resection, but otherwise remained clinically stable. Surveillance MRI demonstrated no progression of the residual sacral mass, confirming stable local disease without evidence of metastatic spread. These findings suggest favorable oncologic control (Figure 1b).

## Discussion

We reported a case of chordoma, the most common primary malignant bone tumor of the sacrum [2], arising from the notochord remnants [1-3]. During normal fetal development, the notochord undergoes near-complete regression, with only microscopic cellular remnants persisting within the nucleus pulposus of intervertebral discs. These vestigial notochordal cells, typically replaced by chondrocytes during early adulthood, represent the putative origin of chordomas when malignant transformation occurs [1].

Primary spinal tumors represent 30% to 40% of neoplastic CES and include chordomas (40%-50%), giant cell tumors (GCTs) (15%-20%), and chondrosarcomas (10%-15%) [1]. Clinical differentiation between these entities is sometimes difficult due to overlapping presentations. Chordomas are the most common primary spinal tumor affecting the sacrococcygeal region, accounting for roughly 50% of cases, and typically affect males aged 30 to 70 years [1,5]. They usually present with chronic midline pain, consistent with their origin from notochordal remnants [1].

Chordomas pose significant diagnostic difficulties due to their indolent growth and nonspecific early symptoms, which often mimic degenerative spine disorders, leading to frequent diagnostic delays [5]. Despite their indolent growth pattern, chordomas demonstrate clinically aggressive behavior marked by high recurrence rates [6] and triplanar invasion across osseous, neural, and visceral compartments. This invasive potential results in a progressive clinical spectrum that often begins with nonspecific mechanical back pain, frequently misattributed to degenerative etiologies, and advances to neurologic deficits such as radiculopathy or CES, vesicosphincter impairment, and bowel involvement [1,2,6].

Sacrococcygeal chordoma is typically characterized by its midline-crossing lytic, expansile lesion on imaging. Still, its features overlap with chondrosarcoma, GCTs, and metastatic lesions to the sacrum [2,7,8]. MRI, particularly T2-weighted sequences, is superior for visualizing soft tissue invasion and neural involvement, while CT excels at delineating bony destruction. Immunohistochemical analysis is necessary to confirm the diagnosis and exclude mimics [1,9].

Chondrosarcomas are more likely to appear off-midline, usually affecting the lateral sacrum. MRI typically shows an intermediate T2 signal, and CT imaging often reveals “ring-and-arc” calcifications. These are characteristics of the cartilaginous matrix produced by malignant chondrocytes arranged in lobules with calcified peripheries [10]. GCTs are generally present in younger patients, between 20 and 40 years of age, and are distinguished by rapidly progressive pain and a high risk of pathological fractures. Radiologically, GCTs often show a “soap-bubble” appearance, which reflects expansile lytic bone destruction [11]. Unlike chordomas, both chondrosarcomas and GCTs usually lack pronounced T2 hyperintensity due to the absence of a myxoid matrix [7,10,11].

While nuclear brachyury expression remains the gold standard due to its near-complete specificity for chordoma [9,12,13], definitive diagnosis in resource-limited settings can be achieved through a triad of characteristic findings: (1) histomorphologic identification of physaliphorous cells (vacuolated cytoplasm within lobulated myxoid stroma), (2) classic MRI features (midline destructive sacral lesion with marked T2 hyperintensity and heterogeneous enhancement), and (3) immunohistochemical co-expression of pan-CK and S100 protein.

Key differential diagnoses include chondrosarcoma (S100+ but typically pan-CK-), metastatic adenocarcinoma (pan-CK+ but S100-, except for melanoma, which is SOX10+), and GCT of bone (lacks physaliphorous cells and shows osteoclastic giant cells) (Table 1) [1,13]. In the absence of brachyury, careful exclusion of these mimics, supported by clinical-radiologic correlation, maintains diagnostic accuracy when advanced molecular testing is unavailable [12,13].

**Table 1:** Comparison between chordoma, chondrosarcoma, giant cell tumor, and metastatic adenocarcinoma MRI: magnetic resonance imaging; CT: computed tomography; pan-CK: pan-cytokeratin; IDH1/2: isocitrate dehydrogenase 1 and 2; RANKL: receptor activator of nuclear factor kappa-Β ligand; PSA: prostate-specific antigen

Tumor Type	Chordoma	Chondrosarcoma	Giant Cell Tumor	Metastatic Adenocarcinoma
Typical location	Midline (sacrum)	Off-midline (sacrum)	Midline or lateral	Variable
Age (years)	30-70	40-70	20-40	-
MRI features	T2 hyperintense, heterogeneous enhancement	T2 hyperintense (lobulated), heterogeneous "ring-and-arc" calcifications	T2 intermediate, hypointense on T1, expansile	Variable (T2 hypo/hyperintense)
CT features	Lytic, expansile, minimal calcification	"Ring-and-arc" calcifications	"Soap-bubble" appearance, no matrix	Lytic/sclerotic, may be destructive
Immunohistochemistry profile	Brachyury+, S100+, pan-CK+	S100+, pan-CK-, IDH1/2 mutations (in 50-80%)	S100-, pan-CK-, osteoclastic giant cells (RANKL+)	pan-CK+, S100- (site-specific markers, e.g., PSA for prostate)

The treatment paradigm for localized chordoma prioritizes en bloc R0 resection, achieving 65% five-year survival, though 30-50% local recurrence persists due to infiltrative growth [1,5,7]. While traditionally radioresistant, margin-positive (R1/R2) or unresectable tumors benefit from adjuvant proton therapy (50-70% five-year local control vs. conventional radiotherapy) [6,7,9]. Our sphincter-preserving R1 resection reflects current evidence showing comparable oncologic outcomes to R0 resection when combined with adjuvant proton therapy, while better preserving function [14]. While therapeutic options remain limited, several emerging targeted therapies demonstrate promising activity in advanced chordoma, including tyrosine kinase inhibitors, brachyury vaccines, and PI3K/AKT inhibitors [15].

While stereotactic radiosurgery was not performed for our patient, several studies showed that this therapeutic option leads to durable local control and symptom relief with acceptable rates of toxicity when used alone and as an adjunct to surgery. It is also effective for patients with recurrent disease who seek noninvasive treatment or those who are not candidates for resection [16]. Surveillance requires pelvic MRI every three to six months (five years) and chest CT for pulmonary metastasis screening.

## Conclusions

Chordomas, though rare, require a high index of suspicion in patients with persistent midline pain and neurological deficits. Early MRI is essential to avoid diagnostic delays and guide surgical planning. Definitive diagnosis typically relies on immunohistochemical markers, especially brachyury, along with pan-CK and S100 positivity; however, brachyury testing was not available in our case. While en bloc resection with adjuvant radiotherapy is recommended in the literature for optimal local control, our patient underwent function-preserving R1 resection without adjuvant therapy and remains clinically and radiologically stable at 24 months. Given the risk of recurrence, lifelong surveillance with regular imaging is strongly advised.
